# Quantitative assessment of Benzalkonium residues in abattoir wastewater and evaluation of disinfectant application practices in Western Thailand

**DOI:** 10.5455/javar.2025.l971

**Published:** 2025-12-25

**Authors:** Suppada Kananub, Prakorn Jala, Tepyuda Sritrakul, Maneenooch Khiao-In

**Affiliations:** 1 Department of Veterinary Public Health, Faculty of Veterinary Medicine, Kasetsart University, Nakhon Pathom, Thailand; 2 Kamphaeng Sean Veterinary Diagnostic Center (KVDC), Kasetsart University, Nakhon Pathom, Thailand; 3 Department of Anatomy, Faculty of Veterinary Medicine, Kasetsart University, Bangkok, Thailand

**Keywords:** Benzalkonium chloride, abattoir wastewater, disinfectant use, occupational safety, Thailand

## Abstract

**Objective:**

To assess benzalkonium chloride (BAC) contamination in abattoir wastewater and evaluate disinfectant usage practices in Western Thailand.

**Materials and Methods:**

Wastewater samples and questionnaire data were collected from 20 pig and poultry abattoirs. BAC analogues were detected using ultra-high-performance liquid chromatography with diode array detection. Associations between abattoir scale and safety practices were analyzed using Fisher’s exact test, with effect sizes expressed as Cramer’s V.

**Results:**

BAC residues were identified in all abattoirs, including 40% that reported not using BAC-based disinfectants. Four large-scale facilities discharged wastewater into public waterways, posing environmental risks. Significant associations were found between abattoir scale and safety measures: safety training (Cramer’s V = 0.59), personal protective equipment availability (0.57), and spill kit preparedness (0.89). Large-scale facilities adhered to these measures, while small and medium operations showed limited compliance. Although smaller facilities did not discharge into public areas, insufficient safety practices may contribute to BAC accumulation.

**Conclusion:**

BAC residues are widespread in abattoir wastewater. Strengthened regulatory oversight, systematic monitoring, and routine safety training are essential to mitigate environmental and occupational hazards.

## Introduction

Benzalkonium chloride (BAC), commonly known as alkyl benzyl-dimethylammonium chloride (BACs), is one class within the group of quaternary ammonium compounds (*QAC)*. BACs are cationic surfactants extensively used for decontamination in hospitals, livestock farms, and food processing facilities [1,2]. Despite their widespread application, BACs exhibit limited effectiveness against specific microbial species, raising concerns about bacterial resistance. Direct human exposure to BACs has been linked to varioushuman health risks, including pulmonary disorder, immune disorder, and cardiovascular disease [3,4]. Furthermore, chronic toxicity associated with BACs is a significant issue, affecting both human health and aquatic ecosystems [3,5].

The persistence of these disinfectants in the environment has led to the emergence of bacterial resistance to *QAC* [2,4]. Studies have reported the potential transfer of resistance from disinfectants to antibiotics. Although the precise mechanisms of this resistance shift remain unclear, the presence of *QAC* genes has been identified as a contributing factor at the genetic level [6,7].

Wastewater treatment systems in slaughterhouses can reduce BAC levels; however, complete elimination remains unattainable. BAC residues tend to accumulate in sludge and contaminate water bodies, with their persistence being influenced by the alkyl chain length of the compounds. The accumulation of BACs in the environment further exacerbates bacterial resistance [8,9]. In Thailand, there are no reports on BAC contamination in slaughterhouse effluents, and safety awareness and disinfectant usage practices within these facilities have not been documented.

This study aims to investigate the levels of BACs present in wastewater collected from the final treatment pond of slaughterhouses and to examine the practices adopted by abattoirs regarding disinfectant usage.

## Materials and Methods

### Ethical approval

Ethical approval was not required for this study, as it does not involve sensitive personal data or animal subjects.

### Sample collection

Slaughterhouses located in Ratchaburi, Kanchanaburi, and Nakhon Pathom provinces (Western Thailand) were included in this study. A preliminary expert interview indicated that the primary disinfectant used in these facilities was BAC. Commercial disinfectant products in the area typically contain BAC variants with alkyl chain lengths of 10, 12, 14, 16, and 18 carbon atoms (BAC10–18). Consequently, this study analyzed BAC residue in slaughterhouse wastewater.

A total of 20 pig and poultry abattoirs from the three provinces were included for sample collection and administration of questionnaires. The survey respondents were selected using a purposive sampling technique, specifically targeting officials responsible for hygiene and safety management in slaughterhouses. All participating facilities provided informed consent for sample collection and data gathering. One liter of wastewater was collected from the final treatment pond at each facility using polyethylene bottles containing sodium thiosulfate. Samples were stored in cool, dark conditions during transportation to the laboratory at the Faculty of Veterinary Medicine, Kamphaeng Saen Campus. All samples were processed within 48 h of collection. All samples were collected during the summer to reduce the seasonal variation.

### Sample preparation

Before the extraction, wastewater samples were filtered to remove debris. Solid-phase extraction was employed to isolate BAC residues using ProElut™ PWC 150 mg/6 ml cartridges (Dikma Technologies Inc., CA, USA). The extracted analytes were subsequently reconstituted in acetonitrile. Standards of BAC10 and BAC18 were randomly added to 13 samples. The BAC10 standard was spiked during the extraction step, whereas BAC18 was added before nitrogen drying. All samples and standard solutions were filtered through a 0.3 µm nylon filter before freezing at −20°C until analysis.

### Sample analysis

BAC residues were analyzed using ultra-high-performance liquid chromatography with diode array detection, equipped with a ZORBAX Eclipse Plus C18 column (Agilent Technologies, Inc., CA, USA). Chromatographic conditions for BAC analysis are presented in Table 1. A predictive calibration curve was established using standard BAC variants (BAC10–18; LGC Labor GmbH, Augsburg, Germany).

**Table 1.** Conditions for BAC analysis.

Parameter	Condition
Column	ZORBAX Eclipse Plus C18
Phase A	0.3% Formic acid with 25 mM ammonium acetate
Phase B	0.2% Formic acid in Acetonitrile
Gradient (Phase A: B)	20:80
Flow rate	0.15 µl/min
Temperature	40°C
Wavelength	264 nm
Injection volume	5 µl

### Data analysis

Abattoirs’ data were categorized according to their operational capacity, following the recommendations of the Department of Livestock Development (DLD), as presented in Table 2. All abattoir sizes are required to meet the exact foundational requirements of DLD for initiating operations. DLD will strategically apply the size-based classification to identify target groups, thereby enabling tailored support and fostering advancement toward higher-tier standards.

Farm-level data from questionnaires and interviews, along with residual BAC levels, were reported descriptively. Associations between farm-level practices were analyzed using Fisher’s exact test due to the small sample size, with effect sizes expressed as Cramer’s V. Statistical significance was set at *p* < 0.05. Data analysis was performed using STATA 13 (StataCorp LLC., TX, USA).

**Table 2.** Operational capacity for pig and poultry abattoir.

Animals	Operational size (animal per day)		
	Small	Medium	Large
Pig	1–20	21–200	>200
Poultry	1–200	201–4,000	>4,000

To ensure method accuracy, linear calibration curves were generated for each analysis. The correlation coefficient (*r*) of each curve was used to assess the model’s fit, with acceptable values set at *r* ≥ 0.995. Additionally, the recovery rates of BAC10 and BAC18 were evaluated to determine potential analyte loss during extraction, with adequate recovery percentages ranging from 70% to 130%.

## Results

### Method validation

The standard calibration curves for all five BAC forms demonstrated high linearity, with correlation coefficients ranging from 0.996 to 0.998, confirming the reliability of the analytical method. For recovery analysis, the mean concentration of BAC10 was 36.45 mg/l, with an SD of 9.13 mg/l, while BAC18 had a mean concentration of 30.76 mg/l and an SD of 11.40 mg/l. Recovery rates for BAC10 and BAC18 were 72.89% and 102.55%, respectively, supporting the method’s accuracy and reproducibility.

### BAC residue in wastewater

**Figure 1.**
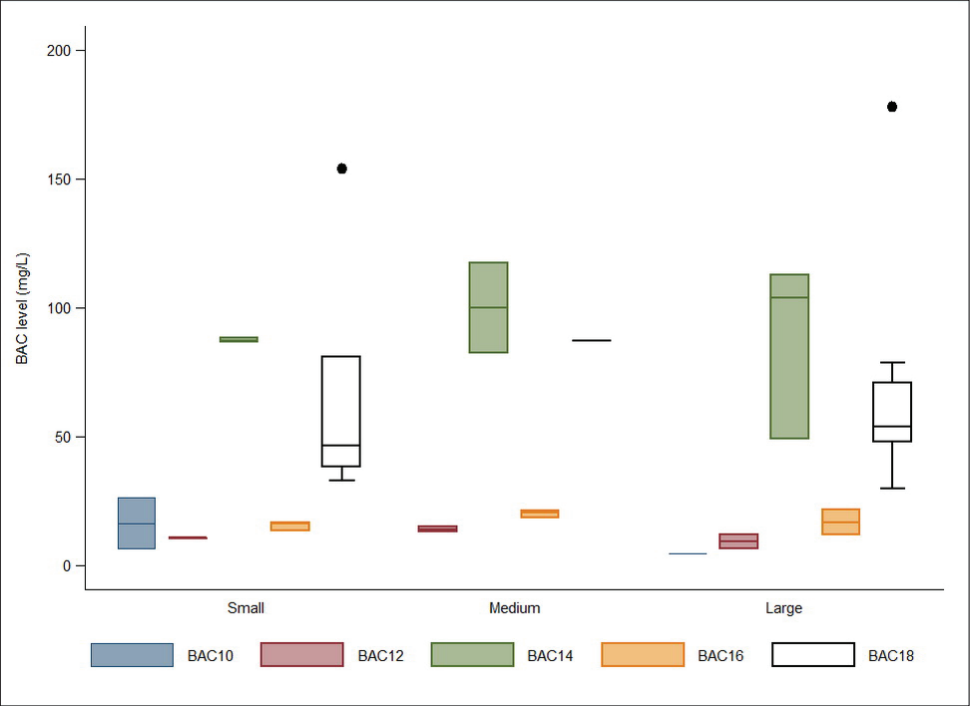
Distribution of residual concentrations of five BAC variants across operational scales.

BACs were detected in all samples collected from the final treatment pond. A total of 12 samples contained a single BAC variant, with BAC16 identified in one sample and BAC18 in the remaining 11 samples. The remaining samples contained multiple BAC forms, with three slaughterhouses detecting a mixture of BAC10 to BAC18. Overall, the residue levels of each BAC type did not differ substantially by operational scale. However, notable patterns were observed. Small-scale slaughterhouses exhibited elevated ranges of BAC14 contamination; medium-scale facilities showed higher levels of BAC18 residues; and both BAC14 and BAC18 were present at elevated concentrations in large-scale slaughterhouses (Fig. 1).

Unexpectedly, eight slaughterhouses detected BAC residues despite reporting no use of BAC-based disinfectants in their cleaning processes. Although BAC levels in these abattoirs were relatively low, seven of the eight samples contained multiple BAC forms. BAC10, BAC12, and BAC16 were detected at low concentrations. In contrast, BAC14 and BAC18 exhibited higher concentrations, with BAC18 reaching a maximum of 180 mg/l (Table 3).

**Table 3.** BAC contamination levels in wastewater (mg/l).

Carbon	*n*	Min	Median	Mean	SD	Max	IQR	Distribution
10	3	4.42	6.09	12.25	12.15	26.25	21.83	Normal
12	6	6.12	11.28	11.1	2.96	14.92	2.38	Normal
14	7	48.72	88.53	91.39	23.26	117.65	30.82	Normal
16	8	11.46	17.35	17.31	3.78	21.74	6.36	Normal
18	16	29.56	53.70	68.79	41.65	177.96	33.84	Non-normal

mg = milligrams, l = liters, n = number, SD = Standard deviation, IQR = Interquartile range.

### Abattoir practices

Of the 20 abattoirs, 12 processed pigs, with five classified as small-scale, two as medium-scale, and five as large-scale. Among the eight poultry abattoirs, 50% were large-scale facilities, while 25% operated at a small or medium scale. The median operational capacity of pig slaughterhouses was 95 pigs per day, with range of 1 to 1,000 pigs per day. In poultry slaughterhouses, the median throughput was 8,000 chicks per day, with a range of 50 to 300,000 birds per day. The DLD officially registered all abattoirs, but only 45% had additional certifications, such as Good Manufacturing Practice, Hazard Analysis and Critical Control Points, or ISO22000—all of which were held by large-scale operations.

Cleaning practices among abattoirs varied by size. Small and medium-sized facilities often use disinfectants irregularly or improperly, relying primarily on detergent-based cleaning. The large-scale abattoirs consistently applied disinfectants after processing. Small- and medium-scale slaughterhouses that do not use BAC in their post-slaughter cleaning processes commonly employ chlorine or chloroxylenol for disinfection. In contrast, some facilities opt for only detergent without any subsequent disinfection step. Large-scale slaughterhouses that similarly exclude BACs from their cleaning protocols tend to use sodium hypochlorite, chlorine, or peracetic acid as alternative disinfectants.

Moreover, small and medium-sized abattoirs did not independently procure or prepare disinfectants; instead, they relied on government support. Among the 20 surveyed abattoirs, 12 confirmed that their cleaning products contained BAC analogs or *QAC*s. However, disinfectant labels did not specify exact compound ratios, though most products contained 10%–20% BACs. Other active disinfectants used in these facilities included peracetic acid, hydrogen peroxide, sodium hypochlorite, and chloroxylenol. Notably, two abattoirs failed to disinfect their work areas, and discrepancies were observed in two additional facilities where disinfectant use was reported but not directly verified.

Safety practices and personal protective equipment (PPE) protocols also varied across different abattoir scales. Three small-scale facilities lacked PPE for workers handling disinfectants, and although some small and medium-sized abattoirs provided PPE, there were no enforcement protocols in place. In contrast, large-scale abattoirs require the use of PPE. None of the surveyed facilities had spill contingency plans for disinfectants. Some facilities planned to report spills to authorities, while others considered them non-critical. Routine safety training was typically reserved for large-scale abattoirs, while smaller operations relied on irregular, government-led training sessions.

Wastewater treatment systems differed based on operational scale. Small abattoirs utilize adapted pond systems and store treated wastewater within their own agricultural areas. Medium-sized facilities also relied on pond treatment but differed in wastewater management, with half reusing water for non-food processes and the remainder using it for irrigation. Conversely, large abattoirs combined pond treatment with biogas, activated sludge, or wetland systems. Nearly half (45%) of large-scale abattoirs discharge wastewater into public sewer systems, yet BAC residues were not regulated under national wastewater standards.

Statistical analyses revealed significant associations between the abattoir scale and three key safety practices (*p* < 0.05): routine safety training (Cramer’s V = 0.59), PPE availability (Cramer’s V = 0.57), and spill kit preparedness (Cramer’s V = 0.89). All large-scale abattoirs implemented these safety measures, while small and medium-sized facilities showed lower compliance, particularly regarding chemical spill preparation, which was absent in all small abattoirs.

## Discussion

Interviews with experts revealed that *QAC*s were the primary substances used for cleaning processes in the study area. However, observational data revealed that BACs, a subset of *QAC*s, were predominantly utilized. Regulatory authorities, such as the U.S. Food and Drug Administration and the Ministry of Industry, have approved BACs for applications in commercial healthcare products, meat processing plants, herbicides, and insecticides [10,11]. The increased consumption of BACs over the past decade has raised concerns about environmental contamination and potential risks to human and animal health [9,12,13].

BACs comprise homologous compounds with alkyl chains ranging from C8 to C18, with variations influencing their solubility and adsorption properties [14]. Short-chain BACs exhibit greater solubility in water, whereas long-chain BACs tend to adsorb onto sludge, thereby increasing their environmental persistence. BACs with alkyl chains of C12–C14 possess the highest biocidal activity and are therefore the dominant homologs in disinfectant formulations [7,15]. In this study, BAC18 was detected in 80% of wastewater samples, indicating its extensive use. The presence of long-chain BACs in treated water suggests a high rate of application, reinforcing the likelihood that most disinfectants in the study area contain BAC18 as a key component. Additionally, BAC14 and BAC18 were identified at notably high concentrations, aligning with contamination levels reported in China, where BAC residues in sediment ranged from 0.09 to 191 μg/gm [13].

The inconsistent disinfection practices observed in small slaughterhouses might not be a result of negligence but rather stem from a lack of knowledge and understanding regarding the necessity of disinfectants and the proper methods of their application. Several studies have demonstrated a correlation between workers’ expertise and good hygienic practices [16,17]. Training slaughterhouse personnel not only ensures the safety of meat for consumers but also improves worker safety and environmental protection in the proper handling of disinfectants [18,19]. Small and medium-sized slaughterhouses require safety training, which may fall under the responsibility of the government [17].

Although 40% of abattoirs reported no use of BAC-based disinfectants, BAC residues were nonetheless detected in their wastewater samples. This discrepancy may result from cross-contamination through sources other than disinfectants [9,20]. According to the U.S. Environmental Protection Agency, approximately 50% of registered *QAC*-based disinfectants contain BAC analogs, while the remainder comprises mixtures of BACs and DADMACs. Given the widespread presence of BACs in household and industrial products, the possibility of unintentional contamination cannot be overlooked. Industries typically inform workers only about general cleaning and hygiene protocols, which may contribute to inconsistencies between workers’ understanding and actual residue findings [9,14,20].

Additionally, the presence of BACs detected in this study may be attributed to residual environmental contamination. Previous reports have identified BACs in various water sources, including surface water, rainwater, and stormwater. BACs in wastewater may be leached from other contaminated sources, contributing to their detection even in locations where use of BACs has not been documented [21,22]. However, this study did not collect data on products other than surface cleaning agents, and no definitive assessment was conducted regarding the sources of residues in abattoirs where BACs were not used.

Concerns regarding BAC toxicity have gained prominence, particularly in relation to human and aquatic health [14]. BACs have been identified in human blood and milk, with prolonged exposure linked to reproductive dysfunction [5]. This substance adversely affects reproductive hormone regulation and spermatogenesis following exposure in a murine model [1]. BACs not only exhibit direct cytotoxic effects but also enhance the absorption of other harmful substances by compromising cell membrane integrity [23]. The inhibitory concentration (IC50) of most quaternary compounds is below 1 mg/l, suggesting significant toxicity even at low concentrations [15]. Given their low biodegradability, BACs pose a long-term environmental risk [14]. Findings from this study indicate BAC levels exceeding toxic thresholds, underscoring the need for regulatory intervention.

Another primary concern is the potential development of antimicrobial resistance (AMR) associated with BAC contamination [4]. Resistance to disinfectants has been linked to the presence of resistant genes, which confer cross-resistance to antibiotics such as kanamycin, gentamicin, tobramycin, and amikacin [9,24]. The precise resistance mechanisms remain unclear. However, evidence suggests that co-localization of resistance genes for BACs and various antibiotics on the same plasmid region facilitates horizontal gene transfer among bacterial populations, thereby accelerating the spread of multidrug resistance. Concurrently, the upregulation of efflux pumps exacerbates resistance by releasing both BACs and antibiotics from the bacterial cell, resulting in reduced intracellular concentrations and diminished susceptibility [6,24,25]. The growing evidence of BAC-related AMR highlights the urgent need for further investigation [6,26].

Although only 20% of sampled slaughterhouses discharged wastewater into public water sources, large-scale facilities generated substantial effluent volumes, contributing to environmental contamination. BACs can be partially degraded through wastewater treatment. However, approximately 8% of quaternary compounds persist in treated water, and approximately 50% accumulate in sludge [2,27]. Some slaughterhouses repurpose the treated water for non-food applications, raising concerns about potential transmission of AMR to workers. Similar contamination issues have been reported in China and Japan, where BAC contamination has declined following stringent government control measures [12].

Findings also highlight significant disparities in safety practices among abattoirs of different operational scales. Small slaughterhouses demonstrated lower compliance with routine safety training, PPE use, and spill kit preparedness, increasing the risk of environmental contamination. Without adequate awareness and training, improper disinfectant usage can unintentionally contribute to pollution. Consistent official training programs are essential to ensure compliance with safe practices. Previous studies have shown that training for new employees in abattoirs often focuses solely on basic cleaning procedures without addressing the details of disinfectant handling [20]. The current findings reveal that 15% of workers failed to wear PPE, with approximately 80% of compliant workers acquiring protective equipment independently. These gaps highlight the need for enhanced regulatory oversight to improve environmental safety and worker protection.

## Conclusion

BAC residues were detected in all samples, with contamination levels notably high. While only large-scale abattoirs discharged wastewater into public waterways, the substantial volumes of effluent released contributed to significant environmental contamination. Given the relatively low acute toxicity of quaternary compounds, chronic exposure to BAC residues may pose long-term risks to both human health and aquatic ecosystems. Monitoring and preventive measures should be implemented to mitigate these risks before contamination reaches an irreversible state. Safety practices among abattoirs varied according to business size, with larger facilities demonstrating greater adherence to safety training, PPE availability, and preparation of chemical spill kits. In contrast, small-scale slaughterhouses exhibited lower compliance, increasing the likelihood of unintentional environmental contamination. Although small and medium-sized slaughterhouses did not directly release wastewater into public areas, their inadequate safety measures may still contribute to the progressive accumulation of BAC over time. Therefore, slaughterhouse operators should prioritize worker protection by implementing routine safety practices to reduce occupational and environmental risks. Further studies may develop learning models tailored to abattoirs of all sizes, fostering sustainable knowledge. Additionally, monitoring contamination levels, assessing their effects, and evaluating regulatory measures remain critical concerns for both human and animal health. In extending the findings of this study to similarly structured slaughterhouses, regulatory bodies should prioritize oversight and support for small- and medium-scale operations. All personnel must undergo training on the use and handling of disinfectants before engaging in any activities related to their use. These groups require targeted and sustained efforts in knowledge management. Additionally, enforceable standards for permissible disinfectant residue levels in effluents should be implemented, together with ongoing monitoring of adjacent water sources to minimize ecological impact.

## List of abbreviations

AMR, antimicrobial resistance; BAC, Benzalkonium chloride; DLD, Department of Livestock Development; IQR, Interquartile range; PPE, Personal protective equipment; SD, Standard deviation; l, liters; mg, milligrams; *r*, Correlation coefficient; µl, microliters; gm, grams.
